# Pandemic Impact on Research Faculty in Academic Medicine: A Mixed Method Study

**DOI:** 10.1089/whr.2024.0091

**Published:** 2025-01-08

**Authors:** Kimberly Bloom-Feshbach, Vasilia Vasiliou, Lance D. Laird, Janet T. Civian, Linda H. Pololi

**Affiliations:** ^1^ Section of Hospital Medicine, Department of Medicine, Weill Cornell Medicine, New York, NY USA.; ^2^ National Initiative on Gender, Culture and Leadership in Medicine: C-Change, Institute for Economic and Racial Equity, The Heller School for Social Policy and Management, Brandeis University, Waltham, MA, USA.; ^3^ School of Business, Clark University, Worcester, MA, USA.; ^4^ Department of Family Medicine, Boston University School of Medicine, Boston, MA, USA.

**Keywords:** career, faculty, gender, pandemic, researchers, women

## Abstract

**Introduction:**

This mixed-method study sought to elucidate the impact of COVID-19 on the professional environments and career trajectories of midcareer research faculty in U.S. medical schools.

**Methods:**

Participants were 40 midcareer medical school faculty enrolled in the Brandeis University C-Change Mentoring and Leadership Institute, a group peer mentoring career development course being tested in a National Institutes of Health-funded randomized controlled trial.

**Results:**

We observed a gender disparity in both the quantitative and qualitative data, with women faculty describing COVID-19 more negatively impacting their career trajectory. This negative impact was independent of having children in the home. Participants largely reported no change in their commitment to conducting research or interest in applying for research funding. A total of 54% of faculty reported no effect of the pandemic on their relationships with colleagues (*n* = 21) and 33% reported a negative effect (*n* = 13). A trend emerged when examining the data by degree, however, with PhD faculty about twice as likely as physicians to report a negative effect of the pandemic on their relationship with colleagues (47% *n* = 9 vs. 20% *n* = 4, respectively). The ordinal test on the 5-point scale approached statistical significance but did not meet the standard 0.05 cut-off (*p* value = 0.06; Z-value = −1.86).

**Conclusions:**

While faculty initially reported some positive outcomes of the SARS-CoV-2 pandemic in their own experiences in April 2020, their experiences 1 year later reflected negative impacts of the pandemic on career trajectory, especially for women, and on relationships with colleagues, with a higher intensity signal for PhD scientists.

## Introduction

Despite challenges during the COVID-19 pandemic, some medical school faculty reported positive outcomes of the pandemic in their own experiences in April 2020, such as increased meaningfulness of work, a reassertion of professionalism and moral responsibility, and enhanced relationships with colleagues.^1^ As described in our earlier report, laboratory shutdowns deeply impacted research faculty. While some faculty appreciated this time and found enhanced productivity in writing, most expressed frustration at the constraints in their inability to conduct experiments, which led to unforeseen interruptions and delays. In addition, many clinicians were called upon to increase patient care duties to meet the needs of the COVID-19 surge in patient volume. As the initial surge of COVID-19 evolved into a protracted global phenomenon, we sought to better understand how COVID-19, 1 year later, was impacting the work and lives of medical school research faculty, on whom society depends for medical research, education, and advancing science.

## Methods

We conducted a mixed-methods study in the context of a randomized controlled trial (RCT) to test the efficacy of a career development program for mid-career research faculty. Quantitative survey data were collected in the fall of 2020. Qualitative data were collected in April 2021.

### Sample

Participants were 40 early midcareer medical school faculty enrolled in the Brandeis University C-Change Mentoring and Leadership Institute, a group peer mentoring career development course being tested in a National Institutes of Health (NIH)-funded RCT.^2^ The faculty sampling frame was primarily the NIH RePORTER database, from which all U.S. medical school faculty who had received recent K, R01, or R01-equivalent awards were invited to apply.

The research design called for stratification of 40 participants by race and ethnicity, gender, and degree (MD or MD/PhD vs. PhD). Using NIH categorization, faculty belonging to racial and ethnic groups that have been underrepresented in medicine (URM) included American Indian or Alaska Native, Black or African American, Latinx/Hispanic, and Native Hawaiian or other Pacific Islander. Faculty belonging to groups not underrepresented in medicine (non-URM) included Asian and White non-Hispanic faculty. Of 270 applicants, 99 met the inclusion criteria and were stratified and randomly selected for inclusion.

### Quantitative data collection and analysis

In the fall of 2020, we surveyed participants about the effects of the COVID-19 pandemic on their careers. Specifically, participants responded to questions regarding the impact of COVID-19 on their commitment to conducting research and interest in applying for research funding using an anchored five-point scale ranging from “greatly decreased” to “greatly increased.” Two additional questions probed the impact of COVID-19 on their career advancement prospects or trajectory, and participants’ relationships with colleagues using an anchored five-point scale ranging from “very negatively” to “very positively.” Using the ordinal five-point scale, with the Mann–Whitney *U* test^3,4^ or Kruskal–Wallis *H* test in Stata/BE 18.0,^5^ we assessed differences by gender, race and ethnicity, degree (MD or MD, PhD vs. PhD), and whether subjects had children under 18 at home.

### Qualitative data collection and analysis

In April 2021, the principal investigator contacted 40 enrolled faculty by email, inviting them to respond by email to two questions: (1) How has the coronavirus affected the meaning you find in your work? and (2) How are you feeling about your role and career choice now with the SARS-CoV-2 pandemic? Two reminders were sent. For the qualitative analysis, we followed the Standards for Reporting Qualitative Research (SRQR) reporting guideline^6^ with a goal of elucidating the lived experiences of faculty in the spring of 2021, 1 year after the onset of the COVID-19 pandemic, focusing on their perceptions of the personal and professional impact of the pandemic.

Before coding, narrative responses were de-identified and aggregated. To minimize bias, two coders with different disciplinary backgrounds consciously bracketed their own experiences with the COVID-19 pandemic^7^ and derived codes by repeatedly reading the aggregated data to develop understanding without reference to degree or demographic variables. The coders developed a final set of codes by consensus to ensure intercoder reliability.^8–10^ Codes were reviewed by two separate qualitative researchers who did not perform the initial coding. We coded data using Excel 16.50.^11^

Analysis involved data reduction and condensation, from which we identified themes and patterns in the coded data. We used an inductive process, in line with grounded theory.^12^ To verify conclusions, two investigators returned to the aggregated data, reevaluating findings to develop intersubjective consensus and to assess for thematic variation in demographic groups of respondents. Given a gender gap revealed in the quantitative analysis, we present here a thematic analysis of the participants’ responses by gender, to gain insight into the experiential differences between and among faculty identifying as women and men.

## Results

The response rate to the quantitative survey was 100%. By study design, half were women and half from groups historically URM. Slightly more than half (*n* = 21) held an MD or MD and PhD degrees, and 26 (68%) had children younger than 18 years old living at home (Table 1).

**Table 1.** Demographic Attributes of 40 Faculty Subjects Completing the COVID Impact Survey in Fall 2020 (*n* = 40, RR = 100%)

Attribute	Total *n* (%)	Male *n* (%)	Female *n* (%)
Gender			
Male	20 (50)	20 (100)	—
Female	20 (50)	—	20 (100)
Race and ethnicity			
URM	20 (50)	10 (50)	10 (50)
Non-URM	20 (50)	10 (50)	10 (50)
Degree			
PhD	19 (48)	8 (40)	11 (55)
MD and MD, PhD	21 (53)	12 (60)	9 (45)
Children under 18 at home			
Children	26 (68)	16 (80)	10 (56)
No Children	12 (32)	4 (20)	8 (44)

URM, underrepresented in medicine; Non-URM, not underrepresented in medicine.

The response rate on the narrative questions regarding COVID-19 impact was 27/40 (67.5%). Respondents included 12 women (44.4%) and 15 men (55.6%). There were 13 URM (48.1%) and 14 non-URM (51.9%) faculty, 15 physician-investigators (55.6%), and 12 PhD-scientists (44.4%).

### Quantitative results

#### Commitment to research

The majority of participants reported no change in their commitment to conducting research (63%). Twenty percent reported decreased, and 20% increased commitment. Interest in applying for research funding was similarly distributed, with about half reporting no change in their interest and the remainder divided between decreased and increased interest (Table 2).

**Table 2.** Frequency Responses Regarding the Impact of COVID-19 on Participants’ Commitment to Conducting Research, Interest in Applying for Research Funding, and Participants’ Relationships with Colleagues by Degree^a^

	COVID-19 impact on	
	Commitment to conducting research *n* (%)	Interest in applying for research funding *n* (%)
Response		
Decreased	8 (20)	9 (23)
No Change	25 (63)	22 (55)
Increased	7 (18)	9 (23)
Total	40 (100)	40 (100)

^a^ The frequencies are displayed using a collapsed three-level scale, where: “decreased” combines greatly and somewhat decreased, and “increased” combines greatly and somewhat increased.

#### Gender and career trajectory

No statistically significant differences emerged when examining the effect of COVID-19 on career trajectory by race and ethnicity, degree, or having children at home, but career advancement trajectory significantly differed for men and women (Table 3, *p* = 0.04; Z-value = 2.05). Female faculty were much more likely to report a negative effect of COVID-19 on their career advancement than their male counterparts. Examining the frequencies, 60% (*n* = 12) of women reported a very negative or somewhat negative effect of COVID-19 on their career advancement prospects compared to 20% (*n* = 4) of men, who largely responded “no effect.” Very few men or women reported a positive effect of COVID-19 on their career trajectory.

**Table 3.** Frequency Responses Regarding the Impact of COVID-19 on Participants’ Prospects or Trajectory of Career Advancement by Gender and Children at Home Under 18

	Response	Total	Gender		Total	Children	
		*n* (%)	Male *n* (%)	Female *n* (%)	*n* (%)	Children *n* (%)	No children *n* (%)
COVID-19 impact on prospects or trajectory of career advancement	Very negatively	1 (3)	0 (0)	1 (5)	1 (3)	1 (4)	0 (0)
	Somewhat negatively	15 (38)	4 (20)	11 (55)	14 (37)	8 (31)	6 (50)
	No effect	18 (45)	13 (65)	5 (25)	18 (47)	14 (54)	4 (33)
	Somewhat positively	4 (10)	2 (10)	2 (10)	3 (8)	1 (4)	2 (17)
	Very positively	2 (5)	1 (5)	1 (5)	2 (5)	2 (8)	0 (0)

#### Impact of having children at home

While 68% of respondents reported having young children at home, statistical tests indicated no difference in career trajectory between respondents with and without young children or on any of the other three survey questions examined. Subsequent analyses using Kruskal–Wallis *H* tests were conducted to determine if COVID-19’s impact on the four studied measures was different for the four groups: men with or without children at home (*n* = 16 and *n* = 4, respectively) and females with or without children at home (*n* = 10 and *n* = 8, respectively). Kruskal–Wallis *H* tests revealed no statistically significant differences in any of the measures among the four groups, although small cell sizes made differences difficult to detect.

#### Relationships with colleagues

Table 4 displays results concerning COVID-19’s effect on relationships with colleagues. About half of faculty reported no effect, and 33% reported a negative effect. A trend emerged when examining the data by degree, however, with PhD faculty about twice as likely as physicians to report a negative effect of the pandemic on their relationship with colleagues (47% *n* = 9 vs. 20% *n* = 4, respectively). The ordinal test on the 5-point scale approached statistical significance (*p* = 0.06; Z-value = −1.86).

**Table 4.** Frequency Responses Regarding the Impact of COVID-19 on Participants’ Relationships with Colleagues by Degree

			Degree	
	Response	Total *n* (%)	MD Total *n* (%)	PhD Total *n* (%)
COVID-19 impact on relationships with colleagues	Very negatively	2 (5)	1 (5)	1 (5)
	Somewhat negatively	11 (28)	3 (15)	8 (42)
	No effect	21 (54)	12 (60)	9 (47)
	Somewhat positively	3 (8)	3 (15)	0 (0)
	Very positively	2 (5)	1 (5)	1 (5)

#### Qualitative results

We analyzed qualitative data by gender, race and ethnicity, and MD versus PhD status. Given the gender gap demonstrated by our quantitative findings, we focus the presentation of qualitative data on the comparative experiences of the pandemic on research faculty stratified by gender identity. The analyses by race and ethnicity and professional background are not presented due to space constraints.

##### Female Faculty

Some women noted challenges of remote work, isolation, and decreased contact with colleagues and patients. A female faculty member described it as:

“Working remotely and doing research behind a computer without in-person human interactions with colleagues makes it challenging to find purpose in life and work as opposed to having a more interactive job where you meet colleagues and patients and engage in mission-driven non-egoistic activities on a daily basis. That feeling of being in service to humanity is substantially reduced by interacting with people only in small windows on a screen.”

Remote work had both challenges and silver linings. Some female faculty expressed gratitude for their ability to use virtual tools to continue meaningful work and to rely on job security during the economic uncertainty of the pandemic:

“I am extremely grateful for having a job and being able to conduct research in online formats and do find meaning when my papers are published and disseminated and joyful when grants come through so I can keep my job and my research team and continue to do our research work to help patients.”

Another woman expressed, *“I am very grateful for my career choice in the past year as I was not worried about employment and was able to maintain productivity working from home.”*

While several faculty reasserted their career choice with gratitude for the ability to continue research with financial security, one physician-scientist noted funding challenges: *“I am feeling frustrated about the lack of resources available to me … [and] by the amount of time and effort needed to obtain NIH funding.”*

Clinical work was cited as a source of stress and frustration for some female clinicians. One MD explained,

“I still feel frustrated by how health care providers were traumatized by the pandemic. I do not like seeing patients now, because I have to get all decked out in PPE and it is physically uncomfortable and isolating. And because too often patients don’t wear masks properly or fail to disclose COVID-19 symptoms and therefore put other people at risk. I am sick of the stress.”

Women experienced interrole conflict, with competing work and family demands: *“More than ever, being a clinician AND investigator AND parent seems impossible. If things do not improve, there is a good chance I will look outside of academia for my next position.”* A female PhD noted that the conflicts between research and the need to care for dependent parents and children had eased somewhat since the onset of the pandemic:

“It was incredibly hard to navigate the impact of the pandemic alongside a growing lab, a terminally ill parent and three elementary aged children. Today, things have reached a bit of a steady state where I don’t have to take each day ‘one hour at a time’.”

Prominent themes among female respondents were gratitude and perceived meaningfulness of work, despite challenges of competing identities and roles. One participant explained, “*While taking time away from my family is hard at this time, it is rewarding to know it is for something that I find meaningful.”* The pandemic also created clarity and focus, prompting some women to reconsider their priorities and be highly intentional.

“The pandemic has affected everything. It has reduced my tolerance for bullshit and for spending time in activities that do not seem meaningful to me or those which could be done by many other people instead of me. In weeding out some of these activities, I am more mindful about how I spend my time and as a result, am more focused on meaningful activities…those that advance public health.”

##### Male Faculty

As with female counterparts, male faculty expressed enhanced meaningfulness and pride in work. One male PhD faculty explained:

“I feel pride about the work of my colleagues around the world who have mobilized against this significant threat…I am very happy about how much effort went into educating the public about vaccines, viruses and immunology. I feel that my discipline is at the center of attention right now, which makes my work more meaningful somehow.”

Like women, some men described fear and isolation, which they connected to the political environment and lack of social interaction.

Clinical faculty across gender credited clinical care in the pandemic with enhanced meaningfulness of work. One male physician stated, *“In my clinical role, it has been gratifying to be involved in the frontlines taking care of COVID-19 patients.”* As another male MD put it, *“being able to contribute positively to alleviating suffering and ending this pandemic makes our sacrifice worthwhile.”* Others described pride in clinical operations and leadership: *“I felt that my actions were very meaningful in helping keep people out of harm’s way. I also felt very proud of the way we organized our response and what I perceive to be its effectiveness.”*

Men also described increased family prioritization, but some did not associate this with stress but rather a change that enabled groundedness and perspective:

“It has…meant more time with the family, forcing [me] to tilt the work/balance aspect of life. It helped slow down things a bit in the beginning, but it soon led to a new normal and things got hectic again. But it has allowed me to pause every once in a while and try to take a big-picture view without always drowning in the angst of the day-to-day minutiae.”

Some male faculty found unexpected opportunities for career advancement. One credited the pandemic as a positive career boost: “the *pandemic has provided me leadership opportunities and the chance to share my knowledge. So on a professional front has been good for me. I have had opportunities to engage with public, the school and state officials as high as the governor*.” Several male faculty appreciated having the “*career training… to understand, vet, and convey information on most all aspects of COVID,”* and found purpose in translating this data for the lay audience. Others reported increased research productivity and leadership impact, which was a source of meaningfulness:

“Being in the front lines of diagnosing and managing patients with COVID-19 while actively pursuing research on this field has been at the same time very taxing and highly rewarding. In many aspects 2020 has been one of my most productive years. I have initiated my promotion process and have applied - successfully - for [another degree]…to increase my capacity to positively impact our field through leadership.”

Some male faculty felt their combination of research and clinical work was protective against burnout during the pandemic: *“I appreciate not having a 100% clinical job-while it is personally rewarding, a full-time job would be a recipe for burnout. Having the balance of my intellectual activities as a scientist and my humanitarian ones as a physician is a good balance.”*

## Discussion

Some findings of our study were consistent with our findings in the 2020 early pandemic study, including the reassertion of career choice and meaningfulness of work.^1^ However, we did observe changes in research faculty's relationships with their colleagues. The sense of community spirit and solidarity of the early pandemic responses evolved toward more distance and isolation due to remote work in 2021.

We observed a gender disparity in both the quantitative and qualitative data, with COVID-19 more negatively impacting women’s perceived career trajectory. This is consistent with other studies demonstrating disparities in academic productivity by gender, including scholarly publication.^13,14^ Some data has linked this gap to having young children at home.^15,16^ Even before the pandemic, among married physicians, women spent significantly more time per day on household activities and childcare than men.^17^ As such, we initially hypothesized that gender disparity may have been driven by challenges of pandemic parenting, including lack of in-person school and childcare.^18^ In our analysis, however, we did not detect any difference in career trajectory based on parenting status. Rather, both male and female faculty described increased home responsibilities.

### Why then did women faculty—independent of parenting status—suffer a perceived impact on their career trajectory?

While women faculty representation has improved, well-described gender inequities in academia persist, with 64% of men representing tenure track faculty in the United States.^19^ The literature reports several barriers for women in choosing careers in academic medicine, including mentoring gaps, women’s interest in teaching over research, and gender discrimination.^20^ In particular, recent prior work has demonstrated considerable sexual harassment.^21^ In such an environment, women may have received fewer opportunities for advancement and/or perceived threats to their careers on the basis of gender. Pandemic-related changes may have impacted professional opportunities or contributed to a context in which women felt a lack of equal footing compared to male colleagues.

We considered the possibility that differences in fields and professional roles contributed to the gender gap noted in our quantitative analysis. Synergy between participants’ areas of expertise and the COVID-19 pandemic led to particular meaningfulness of work in April 2020.^1^ Therefore, it is possible that gender differences in medical specialty or areas of research focus may have contributed to our findings. However, we did not find a difference when adjusting for MD vs. PhD status in our gender analysis on the perceived impact on career trajectory. In addition, some PhDs were clinicians, and others were not. Unfortunately, we did not have access to this data quantitatively. We hypothesize that the role as a clinician may have impacted pandemic experience for research faculty more so than their degree and thus may have diminished some potential differences between MD versus PhD pandemic experiences.

COVID-19 has been linked to high rates of burnout.^22^ In interviews of physicians leaving their practice in the US, 61.5% were women, citing reasons including a challenging healthcare delivery landscape, leadership/local practice culture, burnout, and work-life integration.^23^ These qualitative data are reflected in global trends: one study in Brazil noted burnout in more than 60% of women physicians.^24^ There is some data to suggest that mental health may have declined more among women in the pandemic.^25,26^

In the literature on the impact of COVID-19 on women scientists, some negative research interruptions were also accompanied by positives—more flexible working hours, expansion in professional skills or experience, and a sense of both inner resilience and adaptability.^27^ More research is needed longitudinally to understand the longer-term positive and negative impact of the pandemic on women research faculty.

While our prior analysis of data from spring of 2020 demonstrated that the pandemic fortified community spirit and enhanced relationships with colleagues, this interpersonal connection seems to have degraded over time, particularly for PhD scientists, who noted a negative impact on their relationships with colleagues. We hypothesize that PhD scientists were more likely than MD counterparts to lack in-person professional interactions during the pandemic. While some PhDs are clinicians, these include mental health providers, who might have been more likely to practice telemedicine. A hypothesized lack of in-person interaction may have led to worse relationships in the workplace.

### Limitations

One limitation of the study was the small sample size which limited our power to detect differences between groups. In addition, our participant sample is composed of NIH-funded faculty researchers. While findings may apply to other investigators, they may not be generalizable to all medical school faculty. We also lacked representation of transgender and nonbinary faculty in this sample, leading to a binary portrayal of the impact of gender on the professional experiences of faculty. In addition, distinct questions were asked in the quantitative and qualitative study arms, leading to different types of findings.

COVID-19 clearly intersected with other axes of marginalization to contribute to the differing impact of the pandemic based on structural determinants of health.^28,29^ Some studies have noted that underrepresented faculty, including those who identify as Hispanic, were more likely to leave academic medicine in the pandemic.^30^ As such, we hypothesized that the pandemic may also have had differential effects on faculty of color in our cohort but did not discern any quantitative differences. Race and ethnicity categories incompletely capture the experiences of faculty of color. Our cohort included faculty of East Asian, South Asian, and Middle Eastern heritage, which may have diminished the effect of underrepresented status differences. The temporal association of the pandemic and the murder of George Floyd, which was followed by a national epiphany regarding the longstanding impact of structural racism, may have increased commitment to conducting research in faculty from all demographic groups. More quantitative and qualitative work is needed to understand differential impacts on URM faculty.

## Conclusion

Despite initial positive silver linings in enhanced research faculty professionalism and meaningfulness of work, COVID-19 also negatively impacted some aspects of career advancement, particularly for women, independent of parenting status. Relationships with colleagues became more strained as the pandemic wore on, with PhD scientists noting the greatest impact. Mentoring interventions may be one tool to address the negative impact on career trajectory that women reported. Group peer mentoring is one modality that integrates both career development and community building and may be particularly effective during exceptionally stressful circumstances such as the pandemic.^2^

## Abbreviations Used

non-URM: not underrepresented in medicine
RCT: randomized controlled trial
URM: underrepresented in medicine
